# Effect of Photoaging on the Structure, Optical Properties and Roughness of One-Shade Composite Restoratives

**DOI:** 10.3390/jfb15090245

**Published:** 2024-08-26

**Authors:** Aikaterini Petropoulou, Maria Dimitriadi, Spiros Zinelis, Maria Antoniadou, George Eliades

**Affiliations:** 1 Department of Fixed Prosthodontics, School of Dentistry, National and Kapodistrian University of Athens, 2 Thivon Str., 11527 Athens, Greece; apetrop@dent.uoa.gr; 2 Department of Biomaterials, School of Dentistry, National and Kapodistrian University of Athens, 2 Thivon Str., 11527 Athens, Greece; mardimit@dent.uoa.gr (M.D.); szinelis@dent.uoa.gr (S.Z.); 3 Department of Operative Dentistry, School of Dentistry, National and Kapodistrian University of Athens, 2 Thivon Str., 11527 Athens, Greece; mantonia@dent.uoa.gr

**Keywords:** one-shade composites, degree of conversion, optical properties, roughness, photoaging, photodegradation, ATR–FTIR, UV–Vis, optical profilometry

## Abstract

The aim of the study was to evaluate changes in the degree of C=C conversion (DC%), chemical structure, optical properties and roughness of one-shade composites before/after photoaging. Τhe one-shade materials tested were Charisma Topaz One (CHT), Clearfil Majesty ES-2 Universal (MES), Essentia Universal (ESU) and Omnichroma (OMN), with G-aenial Anterior (CNA) serving as control. Specimens (2 mm thickness) were prepared and tested for DC% and chemical structure (ATR–FTIR spectroscopy), optical properties (L*a*b*–ΔΕ, translucency parameter–TP, opalescence parameter–OP, contrast ratio–CR and total transmittance–TT by UV–Vis spectroscopy) and roughness (Sa, Sz, Sdr, Sds and Sc by optical profilometry) before and after photoaging (Xe-arc weatherometer). Significant differences were found in DC% between top–bottom surfaces (ESU, OMN before; ESU, CNA after). Photoaging improved DC%, reduced ester peaks implying photodegradation, reduced L* (CHT, OMN, CNA), a* (CHT, CNA), b* (OMN, CNA), TP (all, except for MES), OP (only MES), CR (only MES, but an increase in CNA) and TT (CHT, OMN). OMN, CNA and MES demonstrated ΔΕ > 3.3. Photoaging significantly increased all roughness parameters in all materials, except for MES (Sz, Sdr, Sc) and OMN (Sdr). Although listed in the same group, significant differences were found in one-shade composites before and after photoaging. Several products were strongly affected by photoaging, demonstrating evidence of photodegradation, an increased roughness and color changes exceeding the clinically acceptable levels.

## 1. Introduction

The challenge of mimicking natural tooth esthetics with direct resin composite restorations has been recognized as a highly demanding task, requiring materials with a variety in opacity, color value, hue, chroma and high gloss. To address this issue, resin composite systems have been introduced, employing a series of shades reproducing the optical characteristics of dentin and enamel, opacifiers to efficiently mask discolorations in thin layer applications and stains for individual characterization. These materials, inspired by the stratification tooth build-up steps used in dental technology, offer exceptional esthetic results. However, the procedure is time-consuming, technique-sensitive and requires extensive clinical training. Moreover, by layering and intraoral-curing materials with different consistencies and mechanical properties, the final restoration incorporates many material interfaces, creating structural heterogeneity and interlayer porosity, especially in the posterior restorations [1]. To simplify esthetic treatment modalities, one-shade resin composites have been launched. These materials, mainly based on current resin matrix and particle-filler technology, have been optimized in translucency to augment tooth diffuse reflectivity in the overall color of the restoration. Assuming the absence of discolorations, one-shade resin composites can aesthetically simulate many or even all color shades, using only a one-shade product [2,3].

The first commercially available material of this category was Omnichroma (Tokuyama, Tokyo, Japan), a spherical, unisized (0.26 μm) silica–zirconia particle-reinforced composite with 68 vol% filler dispersed in an aliphatic dimethacrylate resin matrix [4]. The 0.26 μm spherical filler interacts with light, creating a red-to-yellow color which is critical for matching natural teeth. This effect, known as the “structural color phenomenon”, is further accommodated by an increase in translucency after irradiation that enhances restoration–tissue color blending [5]. However, it has been postulated that the color-matching capacity and the color stability after immersion in staining solutions of this product is inferior to multi-shade composites [6].

Several other one-shade composites have been marketed based on similar principles. Their mechanical properties have been found to be material-dependent [7,8]. Moreover, significant changes in the surface roughness and hardness of one-shade composites were pronounced after storage in food-simulating liquids [9]. Finally, the degree of conversion, that has a critical impact on mechanical, chemical, optical and biological properties, was found to be higher in aromatic-free monomer products [10].

The long-term color stability of resin composite restorations is an important factor for their clinical acceptability and longevity. Yet, information on one-shade resin composites resins is insufficient, since such studies are mainly focused on their color-matching capacity and the relevant optical properties, without considering material aging [3,11,12,13,14,15]. Only in a limited number of studies has the effect of aging been investigated, with contradictory findings. For example, thermocycling led to clinically unacceptable color changes in one-shade composite [16]. Nevertheless, once polished, the clinically detectable color changes decreased below the clinical acceptability threshold [17]. On the other hand, while accelerated aging caused color instability in several one-shade products [18], others showed lower color changes below the clinically acceptable threshold value [19].

Roughness is associated with the esthetics (gloss, extrinsic staining capacity), biological performance (plaque retention) and tribological properties (friction, wear, etc.) of restorative surfaces. The roughness of one-shade composites has been mainly investigated before and after finishing/polishing procedures employing amplitude parameters (i.e., Sa, Sz) [20]. However, information on the effect of aging conditions on roughness is limited [21].

The aim of the present study was to evaluate the degree of C=C conversion, structure, optical properties and roughness of one-shade composites before and after photoaging. The null hypothesis was that (i) there are no differences in the properties tested among materials before or after photoaging and (ii) photoaging does not affect the material properties.

## 2. Materials and Methods

The materials tested in the present study and their composition are listed in Table 1. CHT, MES, ESU and OMN are one-shade composites, whereas CΝA is a conventional anterior restorative (A2 shade) used as a control.

### 2.1. Degree of C=C Conversion and Structural Changes

Disc-shaped specimens (Ø: 5 mm, h: 2 mm, n = 5/material) were prepared using plastic molds of the same internal diameter. The molds were placed on microscopic glass slide surfaces covered with transparent celluloid matrix strips, which were placed on a flat surface of ≈80% reflectance. Then, they were filled with the composites, covered with another set of strips and glass slides, pressed to remove the material excess and light-cured for 20 s from the top surface with a LED light-curing unit (Radii Plus, SDI, Bayswater, Australia) a emitting 1450 mW/cm^2^ light intensity at standard mode, as measured with a curing radiometer (Bluephase II, Ivoclar Vivadent, Schaan, Liechtenstein). The specimens were demolded, and the degree of C=C conversion (DC%) was measured on the top and bottom surfaces by attenuated total reflectance Fourier-transform infrared spectroscopy (ATR–FTIR), 10 min after irradiation and storage at 37 °C (dark/50% RH), as follows: The central part of each specimen surface was pressed against the reflective crystal (diamond type III, 2×2 mm) of an ATR accessory (Golden Gate, Specac, Oprington, Kent, UK) attached to an FTIR spectrometer (Spectrum GX, PerkinElmer, Buckinghamshire, Bacon, UK). Spectra were acquired under the following conditions: 4000–650 cm^−1^ wavenumber range, 4 cm^−1^ resolution, 20 scans co-addition, 2 μm depth of analysis at 1000 cm^−1^. Spectra of uncured paste specimens were used as unset controls. For the degree of conversion measurements (DC%), the aliphatic C=C stretching vibrations at 1635 cm^−1^ were chosen as the analytical band (AN), whereas the aromatic C..C stretching vibrations at 1608 cm^−1^ (for MES) or the N–H bending vibrations at 1540 cm^−1^ (for CHT, ESU, OMN and CNA) were selected as the reference band (RF). Quantification was performed according to the following equation:

DC% = 100 × [1 − (ApAN × AmRF/AmAN × ApRF)](1)

where A is the net peak absorbance height of set (p) and unset (m) materials at the analytical (AN) and reference (RF) bands, respectively.

The same specimens, after 5 days storage at the same conditions, were subjected to photoaging in a weathering device (Sunset CPS Plus, Atlas, Mount Prospect, IL, USA), under 310–800 nm wavelength irradiation, 765 W/m^2^ irradiance and 66 MJ/m^2^ daily exposure, at 37 °C for 96 h (4×24 h exposure periods with three 1 h relaxation intervals). Then, ATR–FTIR spectra were recorded again, and the degree of conversion was measured as before.

To evaluate the effect of photoaging in the molecular structure of the materials, normalized spectra of paired specimens (same top surfaces before and after photoaging) were compared to identify changes in the abundance of functional groups. Since ester groups are the most susceptible to photoaging in acrylates [22], the peak height absorbance ratios of the ester groups (1720–1709 cm^−1^) to the corresponding reference bands were used to quantitatively assess any changes at the directly exposed surfaces, within the superficial 2 μm zone probed by the ATR–FTIR analysis.

### 2.2. Assessment of Optical Properties

Disc-shaped specimens (Ø: 14 mm, h: 2 mm, n = 10/material) were prepared as before, using square-shaped (20×20×2 mm) black plastic molds with a central hole of the same diameter. Each specimen was irradiated for 4×20 s (cross-mode) over the glass slide using the LED curing unit. Total transmission (T) and total. reflection (R, 8° angle geometry for specular and diffused components) measurements were obtained from all specimens using black (for R) and white (for R and T) backgrounds, employing a UV–Vis spectrometer (Lambda 35, PerkinElmer, Norwalk, CT, USA) equipped with a 50 mm diameter integrated sphere (RSA-PE-20, PerkinElmer). Measurements were obtained under the following conditions: 380–800 nm wavelength range, 480 nm/min scanning speed, 2 nm slit bandwidth and 2° observation angle. Color coordinates (CIELab and CIExyY systems) were measured in total transmission and reflection modes for all specimens. Then, the specimens were subjected to photoaging, as previously described, and measured again in the UV–Vis spectrometer under the same conditions.

Color differences before and after photoaging (ΔΕ*ab) were calculated using the following equation:

ΔΕ*ab = [(L2* − L1*)^2^ + (a2* − a1*)^2^ + (b2* − b1*)^2^]^½^ (2)

where L2*, a2*, b2* are the values after photoaging and L1*, a1*, b1* the corresponding values before photoaging for each individual specimen, with measurements performed in reflectance mode on a white background.

The translucency parameter (TP) was calculated using the following equation:

TP = [(LW* − LB*)^2^ + (aW* − aB*)^2^ + (bW* − bB*)^2^]^½^ (3)

where LW*, aW*, bW* are the values obtained on white, and LB*, aB*, bB* those on black backgrounds, in reflectance mode per specimen.

For the opalescence parameter (OP), the following equation was used:

OP = [(aT* − aR*)^2^ + (bT* − bR*)^2^] ^½^ (4)

where T and R indicate transmittance and reflectance mode measurements on a white background per specimen.

The contrast ratio (CR) was calculated according to the following formula:

CR = Yb/Yw(5)

where Y is the lightness of the specimens against black (Yb) and white (Yw) backgrounds, according to the CIExyY system.

Finally, the average total transmittance (TT) of the specimens was calculated by measuring the sum of transmission per 2 nm bandwidth and then dividing by 210 (the number of the 2 nm bandwidth sectors within the 380–800 nm wavelength range).

Differences in TP, OP, CR and TT (defined as ΔTP, ΔOP, ΔCR and ΔΤΤ, Δ: after–before photoaging) were calculated for each specimen.

### 2.3. Roughness Measurements

The specimens used for assessment of optical properties were also used for roughness measurements before and after photoaging. Regions of interest were randomly selected at the top surfaces under an optical microscope (DM 4000B, Leica Microsystems, Wetzlar, Germany) at 100× magnification, excluding areas with surface defects assigned to specimen preparation (pores, scratches, etc.). Roughness measurements were performed at the regions of interest, employing an optical profiler (Wyko NT1100, Veeco, Tuscon, AZ, USA) operated in vertical scanning mode, 2% modulation, tilt correction and 41.6× effective magnification (20× Mirau lens, 2× field of view, 148×113 μm^2^ analysis area). For each specimen, three measurements were taken at the top surface, and the mean value was used as representative. The 3D roughness parameters determined were Sa (arithmetic mean height, the absolute values of the surface height deviations measured from the best-fitting plane), Sz (the average difference between the 5 highest peaks and 5 lowest valleys of consecutive sampling measurements), Sdr (developed interfacial area ratio, the percentage difference between the true and the projected surface area), Sds (summit density, the number of peaks per unit area of the surface), and Sc (core void volume, the volume the surface would support from 10% to 80% of the bearing ratio) [23].

### 2.4. Statistical Analysis

The normality and equal variance of the measurements were evaluated by Shapiro–Wilk and Brown–Forsythe tests. Two-way ANOVA was used for the statistical assessment of DC% differences between materials and locations (top–bottom), between the optical (L*, a*, b*, TP, OP, CR, TT) and roughness (Sa, Sz, Sdr, Sds, Sc) parameters before and after photoaging. One-way ANOVA was used to determine the statistical differences in ΔΕ, ΔΤP, ΔOP, ΔCR and ΔΤΤ parameters between materials, before and after photoaging. Tukey tests were used to allocate pairs with significant differences. In cases of failures to meet the normality and homoscedasticity criteria, a non-parametric analysis was used. Finally, t-tests were used for the assessment of the normalized ester peak height changes before and after photoaging, and one-way ANOVA for the percentage differences in the ester peak height between the materials per condition. A Sperman analysis was used to identify correlations between the percentage differences in the ester to reference peak height ratios vs. the percentage differences in DC% (top surfaces), and vs. the percentage differences in roughness parameters. All analyses were performed with SigmaStat 14.1 software (Systat Software Inc., San Jose, CA, USA) at a 95% confidence level (α = 0.05).

## 3. Results

### 3.1. Degree of C=C Conversion and Structural Changes

**Figure 1:**
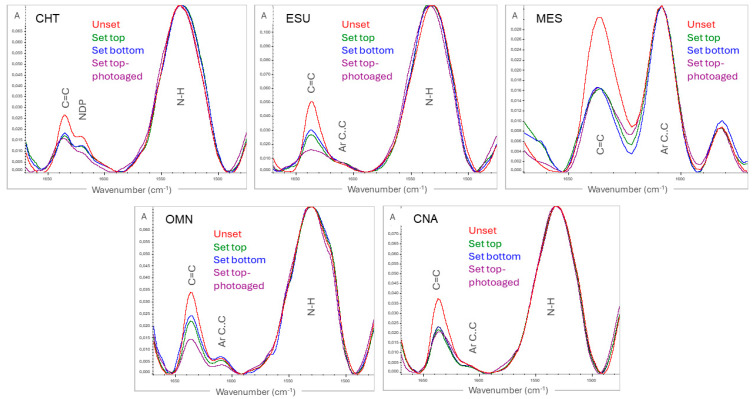
Expanded ATR–FTIR spectra of unset, set and photoaged composite surfaces with the analytical (C=C at 1638 cm^−1^ for all) and reference bands (N–H at 1540 cm^−1^ for CHT, ESU, OMN, CNA and C..C at 1608 cm^−1^ for MES) used for DC% measurements. Note the peak at 1620 cm^−1^ (NDP) in CHT, which is shifted +12 cm^−1^ from the peak of C..C.

ATR–FTIR spectra of unset and set materials (top/bottom surfaces) before and after photoaging, with the characteristic bands used for quantification of the conversion, are illustrated in Figure 1. In all materials, except for MES, the aromatic C..C peak at 1608 cm^−1^ demonstrated very small intensity to be used as a reference band. 

The results of DC% are presented in Table 2. Before photoaging, significant differences between top and bottom surfaces were found in ESU and OMN. At the top surfaces, the ranking of the significant differences was CHT,OMN>CNA,MES>ESU, whereas at the bottom surfaces the corresponding ranking was CHT>CNA,MES,OMN>ESU. After photoaging, top–bottom differences were found in CNA and ESU. The ranking for top surfaces was OMN>CHT,ESU, with MES, CNA demonstrating negligible differences from each of the two groups, whereas for bottom surfaces the ranking was OMN,CHT,MES>CNA,ESU. The analysis of ΔDC% showed a significance ranking of ESU,MES,CNA,OMN>CHT for top surfaces and OMN,ESU>MES>CNA,CHT for bottom surfaces, with significant differences between top–bottom surfaces located in MES, CNA (top) and OMN (bottom).

**Figure 2:**
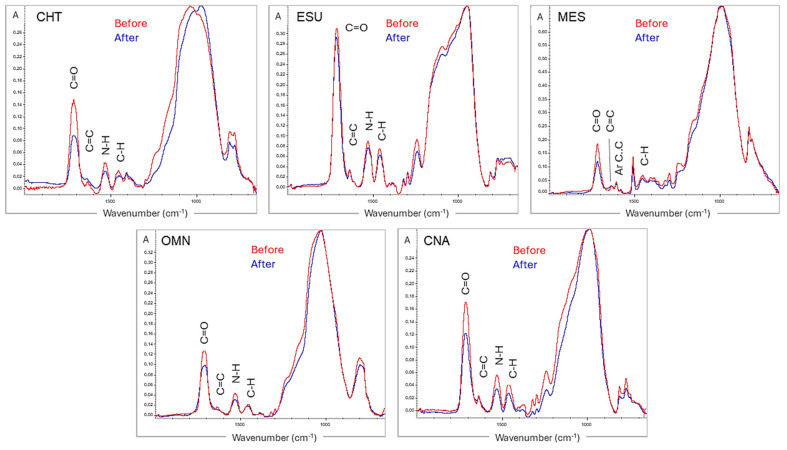
ATR–FTIR spectra of top composite surfaces before and after photoaging. After photoaging, apart from the reduction in the C=C peak (as per Figure 1), the C=O peak demonstrated a strong reduction in some materials (2000–650 cm^−1^ wavenumber range, absorbance scale).

ATR–FTIR spectra of the same top specimen surfaces before and after photoaging at the fingerprint region (2000–650 cm^−1^) are illustrated in Figure 2. The spectra were normalized against the strong and broad complex peak around 1000 cm^−1^ (C=O, C–O–C, C–OH peaks from the resin matrix, and Si–O peaks from SiO_2_ or silicate filler components). In some materials, the C=O peak (1720–1709 cm^−1^) was significantly reduced after photoaging. The results of the C=O to reference band peak height ratios before and after photoaging are presented in Table 3. In all materials, a reduction in the ratio was found after photoaging.

The ranking of the percentage reduction in the normalized ester peak height was CNA,CHT>OMN>MES>ESU, without significant correlation with ΔDC(%)T.

### 3.2. Assessment of Optical Properties

**Figure 3:**
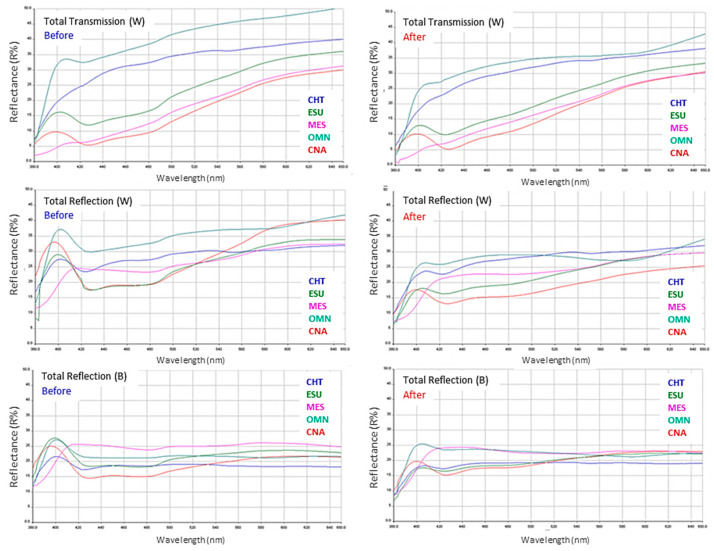
Total transmission and total reflection UV–Vis spectra of the materials before and after photoaging (380–800 nm wavelength range, 0–50 R% units scale, W: white background, B: black background).

Representative UV–Vis spectra of specimens before and after photoaging are illustrated in Figure 3. The total transmittance of MES and CNA (control) were the least affected, contrary to CHT, ESU and OMN, especially at longer wavelengths. The spectra on the white background demonstrated a reduction in reflectance of all materials after photoaging, whereas on the black background the differences were minimized.

The results of the L*, a*, b* color parameters for each material before and after photoaging are presented in Table 4. After photoaging, a significant reduction was observed in L* and a* for CHT, in L* and b* for OMN and in all parameters for the control (CNA). Comparisons among the materials before or after photoaging revealed the following significant differences: OMN,CHT>ESU,MES,CNA (L* before), OMN>CNA, with MES, CHT and ESU demonstrating insignificant differences from both materials (L* after); CNA>ESU,MES>OMN>CHT (a* before), CNA,MES>OMN,CHT, with ESU demonstrating insignificant differences from MES, but significant from OMN and CHT (a* after); CNA>ESU>OMN,MES>CHT (b* before), and CNA,ESU>MES>CHT>OMN (b* after).

The results of the TP, OP, CR and TT parameters are summarized in Table 5. For TP, a significant reduction was observed in all materials after photoaging, except for MES, which demonstrated a significant increase. Before photoaging, the ranking of the significant differences was OMN>CHT,CNA>ESU,MES, while after photoaging the ranking was changed to OMN,MES,CHT>ESU>CNA. For OP, the only significant difference noticed was the reduction in MES values after photoaging, with the ranking of significant differences being MES>CNA>ESU>CHT>OMN before, and MES,CNA>ESU>OMN,CHT after photoaging. Significant differences were registered in CR before and after photoaging in MES (reduction after) and CNA (increase after). Before photoaging, the rankings of the significant differences were MES>CHT,OMN and MES>CNA,ESU>CHT,OMN and CNA>OMN, whereas after photoaging, the significant differences were limited to ESU>OMN and CNA>OMN.

Finally, for TT, significantly reduced values were registered after photoaging in CHT and OMN, whereas the ranking of the significant differences was OMN>CHT>ESU, CNA,MES before, and OMN>CHT>ESU,CNA,MES after photoaging.

**Figure 4:**
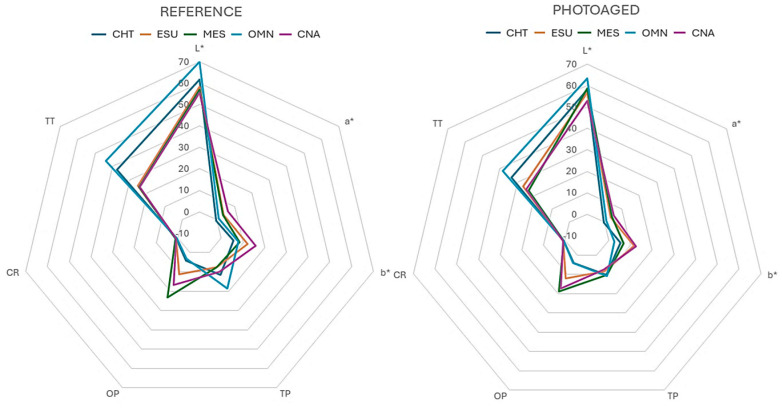
Radar graphs of the optical properties tested before (reference) and after photoaging.

The results of the differences (after–before photoaging) of the optical properties among the materials tested are presented in Table 6. For the color parameters, the ranking of the significant differences was MES>ESU,CNA,CHT>OMN (ΔL*), MES>CHT,OMN,ESU>CNA (Δa*), CHT,ESU>MES>CNA>OMN (Δb*) and OMN>CNA,MES>ESU, with CHT demonstrating insignificant differences from MES and ESU (ΔΕ). The rankings for the other optical properties were ΜΕS>ESU,CHT>CNA>OMN (ΔΤP), OMN>ESU,CNA>CHT>MES (ΔOΡ) and OMN>CNA (ΔTT). The greatest changes in the optical properties were registered in OMN (ΔL*: −6.16, Δb*: −6.59, ΔE: 8.99, ΔΤP: −7.56, ΔΤΤ: −5.4) and MES (ΔOP: −4.34, ΔCR: −0.24). The results of the optical properties tested, before and after photoaging, are summarized in the radar graphs of Figure 4.

### 3.3. Roughness Measurements

**Figure 5:**
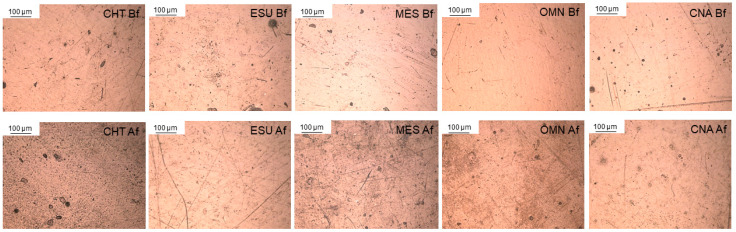
Reflected light microscopic images of top material surfaces before (Bf) and after (Af) photoaging. After photoaging, a micro-pitted structure appeared in several materials (i.e., CHT, MES, OMN; bright field, 100× magnification, bar = 100 μm).

**Figure 6:**
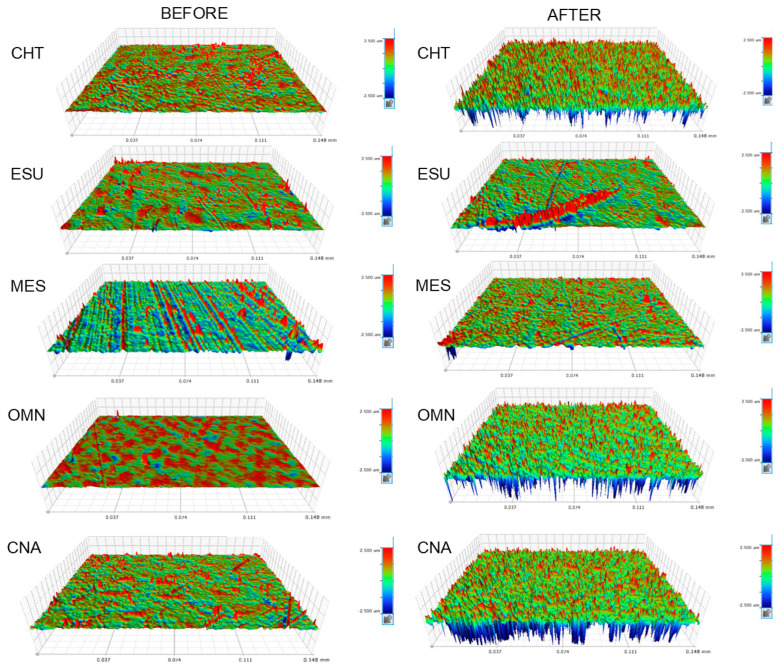
3D-profilometric images of representative surfaces before and after photoaging. A rougher surface texture was evident after photoaging (41.6× magnification, 148×113 μm^2^ analysis area, −2.5 up to 2.5 μm standard amplitude scale).

Reflected light microscopic images of materials before and after photoaging are illustrated in Figure 5. The photoaged materials demonstrated a micro-pitted topography to various extents and distributions. Representative 3D-profilometric images are presented in Figure 6. The surfaces of most materials exhibited increased amplitude deviations after photoaging. The results of the roughness parameters before and after photoaging are presented in Table 7. For all materials, except for MES (Sz, Sdr, Sc) and OMN (Sdr), a significant increase in roughness was registered after photoaging. The ranking of the significant differences before photoaging was MES>CNA,CHT,ESU>OMN (Sa), CNA,MES,ESU, CHT>OMN (Sz), MES>CHT,ESU,CNA>OMN (Sdr), OMN>CHT,ESU,CNA,MES (Sds) and MES>CNA,ESU,OMN>CHT (Sc). After photoaging, the ranking was modified to CHT>ESU,CNA,OMN>MES (Sa, Sds), CHT>ESU,CNA,MES,OMN (Sz, Sdr) and ESU,OMN,CNA>MES>CHT (Sc). No correlation was found between the percentage differences in the ester to reference peak height ratios and the percentage differences in roughness parameters.

## 4. Discussion

The results of the present study showed that there were statistically significant differences between the materials tested in DC%, with structural changes and optical properties before and after photoaging. Therefore, the null hypothesis was rejected.

One-shade dental resin composites have been introduced to simplify shade selection and reproducibility, by effectively blending the restoration color with the surrounding dental hard tissues. This phenomenon, known as the “chameleon effect”, is mainly assigned to increased translucency, defined by the translucency parameter (TP) [24,25]. An increased TP enhances the penetration of the activating light in bulk material, improving the in-depth conversion. However, in two of the materials examined (ESU, OMN), a significant difference was observed in DC% between top and bottom surfaces. For ESU, which contains a bisphenol-A monomer adduct, the results of the present study demonstrated a lower DC% (46.5%) on directly exposed surfaces in comparison with previously reported values (68.5%) [10]. The differences may be attributed to enhanced post-curing reactions (15 days’ storage in water at 37 °C) or to the grinding procedure used to pulverize the samples for transmission FTIR analysis [10]. However, in the same study a much lower conversion was found for the aromatic-free monomer OMN (52%) by using the aromatic stretching vibrations as an internal standard. The ATR–FTIR spectra of OMN (Figure 1) revealed very small peaks of aromatic vibrations at 1608 cm^−1^, probably assigned to other sources than the monomer (i.e., the aromatic photo-initiator), which does not fulfill the requirements for a reliable quantification. The same applies for ESU and CNA, which contain bisphenol-A adducts, apparently in low amounts. For CHT, no aromatic peak was traced at 1608 cm^−1^. Instead, a peak appeared at 1620 cm^−1^ probably assigned to an undefined source of C=C which does not seem to change after light-curing, such as the cyclopentene ring of the synthesis process of TCDU [26], again at low intensity. To overcome these limitations, the N–H vibrations (1540 cm^−1^) assigned to the amide backbone of UDMA were selected as a reference band. At the directly exposed surfaces of ESU, the stiff structure of the BisEMA monomer reduced the DC% in comparison with products without aliphatic monomers, due to steric hindrance phenomena [27]. The same may apply for MES, which yet did not show a significant difference from the control (CNA). At the bottom surfaces, OMN and ESU showed the lowest DC%, with the greatest differences from top. It has been shown that the timeframe of changes in the refractive index of light-cured materials upon irradiation may exceed 100 s [28]. Therefore, the TP of set materials cannot be used to express the light transmission characteristics for the irradiation period. Consequently, the low DC% of ESU at the bottom surfaces, being the least efficiently cured at the top surface, might be explained by a typical in-depth reduction of light intensity due to scattering and adsorption. This is corroborated by the high b* values (yellow shift, 12.2 units) of the material, which absorb the blue activating light. OMN and CHT, demonstrating the highest DC% at the top surface, showed the greatest average DC% reduction (−13.2%) at the bottom surface. Two explanations may be given for this finding: first, the relatively high b* value (8 units) of the material, as discussed above, and second, the excessive light scattering. It has been documented that the maximum scattering of the activating light (peak emission at 468 nm in most light-curing units), which is associated with a reduced DC%, occurs when the filler particle size is about half the wavelength of the activating light [29]. Considering that the filler content of OMN is mainly spherical monodispersed and of 260 nm size to offer a “red-to-yellow structural color” effect [5], an increased light scattering is anticipated for the activating light.

Isothermal storage and photoaging had a positive impact on the conversion of most materials at the top and bottom surfaces. During photoaging, exposure of the materials to wavelengths, including the ranges of peak photo-initiator absorption (i.e., 468 nm for CQ, 395 nm for TPO, etc.) contributed to the expression of a post-curing effect, with an average improvement in DC% of 17.7% at the top and 16.2% at the bottom surfaces. A possible explanation may be given based on the enhanced crosslinking of the matrix after initial exposure, which has long been documented for light-cured resins and composites without [30] and with photoaging [31]. In the present study, photoaging was performed after 5 days’ storage in isothermal conditions (37 °C/dark/dry), which lays within the half-life period of the free radicals produced during photopolymerization and may have contributed to the increased DC% after photoaging [32].

Photoaging of polymethacrylates and aromatic polycarbonates is mainly pronounced at short wavelengths (<300 nm), by the degradation of ester groups and the random fracture of polymer chains in the former, or the formation of phenyl esters and benzophenones in the latter [22]. The mechanism is far more complex in the presence of oxygen (air) or water. Polyperoxides (for polymethacrylates) and low-molecular-weight byproducts such as oligomers, bisphenol-A, ring opening oxidates and cyclic anhydrite units (for aromatic polycarbonates) are released, which are further oxidized much more easily than the polymers. The same applies for the low content of the remaining monomers, which absorb light more than polymers [22]. Visible light (>400 nm, especially the green light at 540 nm) may induce photodegradation as well, but to a milder extent [33]. For composite restoratives, the red/yellow pigments used in many shades absorb the higher-energy short-wavelength light, reducing the penetrating light fraction and thus the intensity of photoaging. The same applies for materials with a low L*, where the absorbed light fraction is increased, protecting the substrate. In contrast, bluish shades (i.e., enamel shades) with a high L* are more prone to photoaging [34]. The ATR–FTIR analysis demonstrated a reduction in the relative intensity of the main ester peak in all products, but at a significantly higher extent in CHT, CNA and OMN, indicating a typical photodegradation effect. Although it is not known if the reference bands (N–H, primary amide of urethane dimethacrylates, or Ar C...C of bisphenol dimethacrylate adducts) are also affected by the specific photoaging conditions used, the greater reduction in ester groups may be explained by the better resonance stabilization of amide and bisphenol bonds. The absence of a significant correlation between the percentage changes in the normalized ester groups (ΔΤ%) vs. the percentage changes in the degree of conversion (ΔDC%) at the top surfaces (T) before and after photoaging indicates that the reduction in the intensity of the ester groups observed was not associated with the post-curing conversion after photoaging. This is important, since a reduction in the C=O intensity occurs after polymerization due to a loss of conjugation with the C=C groups via hydrogen bonding [35]. Consequently, the changes observed should be mostly attributed to the photodegradation process.

The materials tested are classified as one-shade composites. However, there were important differences in their color properties at baseline, with the greatest found in L* (56.9–69.9), a* (−0.6–6.2), b* (5.6–12.2), TP (7.7–18.5), OP (4.1–23.3), CR (0.6–0.9) and TT (24.6–43.9). The optical properties of composite restoratives are mainly controlled by the refractive indices of resin and fillers, the type of monomers, the shape, size distribution and volumetric filler loading, and the presence of internal defects (i.e., pores) [36]. The one-shade materials tested as a group demonstrated in average higher L*, ΤΤ and lower a*, b* values from the control (CNA). This agrees with previous findings on the increased translucency of one-shade composites [11]. OMN and CHT showed the highest L*, TP and TT and the lowest a*, b*, OP and CR values from the group of one-shade composites. The increased TP and TT values agree with the low CR values, since the high transparency of the structure has a reduced masking capacity for the substrate. The lowest nano-hybrid filler content of CHT (56 wt%) and the uniform nano-spherical filler geometry of OMN may facilitate light penetration, since filler size and shape strongly affect light scattering [37]. For ESU and MES, the CR and TP values were, respectively, higher and lower than the control (CNA), which suggests that a rather wide range of translucency values are employed in the design of one-shade composites. These materials demonstrated the highest blue shift, although significantly lower than the control. For OP, which defines the chroma difference between the reflected and transmitted light (a bluish appearance under reflected and orange/brown appearance under transmitted light) [38], the MES values were significantly higher than the control (CNA), followed by ESU (lower than the control), and the group of OMN and CHT, which provided the lowest values. Opalescence is influenced by the difference in the refractive index of the resin matrix and fillers, the amount and shape of fillers, pigments and additives [39]. Two of the one-shade materials tested (CHT, OMN) showed OP values below the range reported for resin composites (5.7–23.7) [39], whereas the CR values of the one-shade composites were within the range registered for composites used for restoring whitened teeth [40].

Photoaging induced significant alterations in all optical properties of the materials tested. A significant reduction was registered in L* (CHT, OMN, CNA), a* (CHT), b* (OMN, CNA), TP (CHT, OMN, CNA), OP (MES), CR (MES, CNA) and TT (CHT, OMN), and an increase in TP (ESU) and CR (CNA) after photoaging. The changes in the color coordinates of resin composite restoratives after photoaging agree with previous results, which demonstrated mild to severe discoloration dependent on the composition of the materials used [41,42,43,44,45,46]. Photoaging is considered as an effective method of testing the intrinsic color stability of composites to UV and visible radiation, whereas the extrinsic one is commonly evaluated after immersion in staining solutions [46,47]. The photoaging of resin composites induces changes in the materials’ chemistry assigned to photobleaching (the consumption of residual colored photo-activators, such as camphoroquinone) [48], the photo-oxidation of reducing amine components and residual C=C bonds, which affect the final color [49]. After photoaging, the number of material groups with significant differences per optical property were reduced in comparison with the control state (before photoaging). Based on the ΔΕ*ab values, clinically (perceptive) unacceptable color changes (ΔΕ*ab > 3.3) were found in OMN (8.99), CNA (4.35) and marginally in MES (3.32), whereas for CHT and ESU the changes were clinically acceptable (1.0 < ΔΕ*ab < 3.3) [50]. OMN demonstrated the highest reduction in TP (ΔΤΡ = −7.56) and ΤΤ (ΔΤΤ = −5.4, along with CHT), and MES the highest reduction in OΡ (ΔOΡ = −4.34). Ιt is not known if the sol–gel procedure of the unisize nanofiller used in OMN is implicated with the instability of the optical properties after accelerated aging, as was documented for the mechanical properties of the material [7].

The optical microscopic images of the photoaged surfaces demonstrated a more irregular topography with an unevenly distributed micro-pitted appearance. Roughness measurements revealed a significant increase in the parameters tested in all materials, except for MES. Considering that roughness is highly associated with gloss [51], it is anticipated that MES might better retain gloss after photoaging. It is interesting that amplitude (Sa, Sz), spatial (Sds), hybrid (Sdr) and functional (Sc) roughness parameters were all affected by photoaging, implying a universal change in surface texture. The changes in roughness confirmed the development of photodegradation reactions, which through complex pathways form volatile monomers (methane, CO_2_ and CO) as end products [22]. The gaseous nature of these byproducts may explain the pitted morphology observed after photoaging. MES, with the lowest roughness changes after photoaging, demonstrated a low ester ratio change in the ATR–FTIR measurements. However, no significant correlation was found between the structural changes, as defined by the percentage differences in the ester to reference peak height ratios relative to the percentage differences in roughness parameters. The increased content of aromatic monomers in MES with a stiff bisphenol backbone and high crosslinking capacity, such as BisGMA, may explain the increased matrix tolerance to photodegradation as indicated by the small changes in roughness at the top surfaces.

In the present study, photoaging was limited to 96 h based on previous findings that the major color changes in photoaged composites were detectable between 72 and 120 h [43]. Moreover, the emitted light was cut off below 310 nm to block highly energetic UV-C (100–280 nm) and UV-B (280–315 nm) radiation, allowing lower-energy UV-A (315–400 nm) radiation to be transmitted to the samples, creating milder and probably more reliable accelerated aging conditions. No water immersion was used to clearly resolve the performance of the polymers, since water absorption is strongly implicated in degradation processes by many mechanisms (monomer release, hydrolytic degradation, plasticization, etc.) with additive effects to photoaging. The CIELab system was used for color measurements rather than the more complex CIEDE2000, since a similar human eye perception of color differences was found for the two formulas [52]. Τhe composite specimens were not polished. Therefore, the flat surfaces were smooth, covered with a resin-rich layer. The thickness of this layer in highly filled composites may extend up to 120 nm, since the interparticle spacing is small and there are hydrostatic constraints in extruding more resin onto the outmost surface by the compression applied on highly packed particles [53]. Although the results obtained may deviate from the clinical analogue of finishing and polishing, smooth specimens with a resin-rich layer have been already used in photoaging studies [54,55,56]. Moreover, this layer exists in the contact points of composite restorations, where the uncured material is pressed against the smooth surfaces of interproximal matrices. Significant differences were encountered in some roughness parameters of the resin-rich composite surfaces before photoaging. It is widely accepted that matrix strips leave a very smooth composite surface, but minor defects (i.e., micro-voids, micro-scratches) cannot be avoided [54], which may affect the results. It seems that the apparent composite viscosity, the wettability of the resinous phase to the matrix strip and the resin extrusion rate to the specimen surface during application may contribute to the surface texture of the resin-rich layer.

The clinical significance of the results of the present study on photoaging should be interpreted with caution. The same applies for the results of the properties for the reference materials’ state (before photoaging). Photoaging is already known to affect the optical properties and degree of conversion of conventional resin composites. However, in the present study it has been shown that it induces photodegradation of the resin matrix by mainly affecting the ester bonds, and it increases several roughness parameters. Further studies, employing more complex environmental conditions (i.e., photoaging plus immersion in water or thermocycling) may provide important information about the chemical changes induced and how these affect the surface qualities of the restorative materials.

## 5. Conclusions

At baseline, significant changes were found in the degree of conversion (top–bottom surfaces) and optical properties among the one-shade composites and the control. Photoaging significantly affected the optical properties of one-shade composites, with unacceptable color changes registered in some materials. Structural changes associated with the increased degree of conversion and degradation of the ester groups of the resin matrix, along with increased surface roughness were observed after photoaging, all typical of a photodegradation process.

## Figures and Tables

**Table 1:** The materials tested and their composition.

Material/Code	Composition *	Manufacturer
Charisma Topaz ONE (CHT)	RESIN: TCDU, TEGDMA, DUDMA, accelerators, initiators. FILLER: Barium aluminum fluoride glass, pre-polymerized fillers, highly discrete nanoparticles (59 wt%, 5 nm–5 μm).	Kulzer GmbH, Hanau, Germany
Majesty ES-2 Universal (MES)	RESIN: BisGMA, hydrophobic aromatic dimethacrylate, hydrophobic aliphatic dimethacrylate, camphorquinone, accelerators, initiators. FILLER: Silanated barium glass, pre-polymerized organic filler, pigments (78 wt%, 0.4–1.5 μm size, including inorganic filler of 40 vol%).	Kuraray Noritake Dental Inc., Okayama, Japan
Essentia Universal (ESU)	RESIN: UDMA, NPGDMA, BisEMA, TCDDMA, accelerators, initiators. FILLER: Barium glass fillers (700 nm), YbF_3_, pigments (81 wt%, 59 vol%).	GC Corp., Tokyo, Japan
Omnichroma (OMN)	RESIN: UDMA, TEGDMA, accelerators, initiators. FILLER: Spherical silica–zirconia filler (79 wt%, 68 vol%, mean particle size: 0.26 μm, particle size range: 0.2–0.4 μm), composite filler.	Tokuyama Corp., Tokyo, Japan
G-aenial anterior (CNA-Control, A2 shade)	RESIN: UDMA, NPGDMA, BisEMA TMPTMA, accelerators, initiators. FILLER: Pre-polymerized fillers. (16–17 μm), strontium glass (400 nm), lanthanoid fluoride (100 nm), silica glass (850 nm), fumed silica (16 nm), YbF_3_, pigments (76 wt%).	GC Corp., Tokyo, Japan

* According to the manufacturers’ information. BisGMA: Bisphenol glycidyl dimethacrylate, BisEMA: Bishenol ethylene glycol diether dimethacrylate (n = 3, 6), DUDMA: Diurethane dimethacrylate, NPGDMA: Neopentylglycol dimethacrylate, TCDDMA: Tricyclodecane dimethanol dimethacrylate, TCDU: Tricyclodecane-urethane dimethactylate (TCD-DI-HEA), TEGDMA: Triethyleneglycol dimethacrylate, TMPTMA: Trimethylolpropane trimethacrylate, UDMA: Urethane dimethacrylate.

**Table 2:** Results of degree of conversion (DC%) at top (T) and bottom (B) specimen surfaces, before (Bf) and after (Af) photoaging, and their percentage difference (ΔDC%) on T and B surfaces (means and standard deviations) *.

Material	DC% T Bf	DC% B Bf	DC% T Af	DC% B Af	ΔDC%T (Af-Bf)	ΔDC%B (Af-Bf)
CHT	62.35 (2.71) a,A	58.33 (1.66) a,A	70.15 (0.73) a,A	68.05 (1.18) a,A	7.8 (2.3) a,A	9.73 (1) a,A
ESU	46.38 (2.1) b,A	39.2 (0.89) b,B	68.8 (1.16) a,A	60.65 (1.35) b,Β	22.43 (2.95) b,A	21.36 (1.2) b,A
MES	52.83 (1.79) c,A	50.63 (1.27) c,d,A	74.15 (2.77) a,b,A	67 (2.38) a,c,A	21.35 (3.27) b,A	16.48 (1.6) c,B
OMN	60.2 (3.77) a,A	47.83 (4.46) d,B	77.05 (1.48) b,A	70.63 (3.96) a,A	16.85 (2.4) b,A	22.7 (2.36) b,B
CNA	53.15 (1.26) c,A	51.65 (1.84) e,A	73.2 (4.9) a,b,A	62.28 (3.73) b,c,B	20.25 (4.6) b,A	10.63 (2.5) a,B

* The same lower-case letters indicate statistically insignificant differences between materials at top and bottom surfaces, before and after photoaging, and their percentage differences per location. The same upper-case letters show the insignificant differences between top–bottom surfaces per material and their percentage differences, before and after photoaging (*p* > 0.05).

**Table 3:** Results of the changes in the normalized peak height of the C=O groups vs. the reference bands (R) at top (T) material surfaces, before (Bf) and after (Af) photoaging (means and standard deviations), and the corresponding percentage mean differences (Δ%) *.

Material	(C=O/R) T Bf	(C=O/R) T Af	Δ%Τ (Af-Bf)
CHT	2.98 (0.18) A	2.5 (0.38) B	−16.1 (1.88) a
ESU	3.98 (0.31) A	3.97 (0.16) A	−0.25 (0.01) b
MES	5.38 (0.3) A	5.12 (0.11) A	−4.83 (0.18) c
OMN	3.21 (0.12) A	2.91 (0.28) A	−9.34 (0.62) d
CNA	3.49 (0.18) A	2.94 (0.56) B	−15.76 (1.9) a

* The same upper-case letters show insignificant differences per material (before and after photoaging) and the lower-case, those among materials in the percentage differences. R: N–H groups for all materials, except for MES, where R: aromatic C..C groups.

**Table 4:** Results of L*, a*, b* color parameters before (Bf) and after (Af) photoaging measured at top surfaces *.

Material	L*Bf	L*Af	a*Bf	a*Af	b*Bf	b*Af
CHT	61.62 (60.21/61.78) a,c,A	58.18 (57.63/59.32) b,B	−0.61 (0.11) d,A	−0.38 (−0.41/0.28) b,B	5.6 (0.3) d,A	5.39 (0.27) c, A
ESU	58.24 (57.61/58.68) d,e,A	56.81 (56.32/57.8) b,A	3.69 (0.2) c,A	3.6 (3.47–3.71) a,A	12.2 (1.32) c,A	11.58 (0.48) a,A
MES	56.9 (54.92/58.48) b,c,e,A	58.63 (56.77/59.62) a,b,A	3.48 (0.59) c,A	4.3 (3.65–5.61) a,A	8 (1.32) a,A	6.82 (2.4) c,A
OMN	69.86 (66.49–71.48) a,A	63.28 (60/64.77) a,B	0.92 (0.31) b,A	0.88 (0.59/1.19) a,A	8.3 (0.72) a,A	2.53 (2.19) b,B
CNA	55.48 (54.66–55.57) b,d,A	52.75 (52.55/53.8) b,B	6.23 (0.32) a,A	5.16 (4.77/5.52) a,A	15.78 (0.58) b,A	12.6 (1.5) a,B

* The same lower-case letters indicate mean values and standard deviations (a*Bf, b*Bf, b*Af) or median values and interquartile ranges (L*Bf, L*Af, a*Af) with statistically insignificant differences among materials, whereas the same upper-case letters show the insignificant differences before and after photoaging per material (*p* > 0.05).

**Table 5:** Results of translucency parameter (TP), opalescence parameter (OP), contrast ratio (CR) and total transmittance (TT), before (Bf) and after (Af) photoaging (means and standard deviations) *.

Material	TP Bf	TP Af	OP Bf	OP Af	CR Bf	CRAf	TT Bf	TT Af
CHT	11.64 (0.7) a,A	10.19 (0.94) a,B	4.09 (0.34) a,A	4.07 (0.34) a,A	0.63 (0.04) a,A	0.71 (0.05) a,b,c,A	37.58 (1.73) a,A	33.27 (1.45) a,B
ESU	7.73 (1.07) b,A	8.25 (1.42) a,B	11.26 (1.23) b,A	11.97 (0.65) b,A	0.89 (0.1) b,c,A	0.87 (0.08) a,b,c,A	25.62 (3.2) b,A	26.58 (1.17) b,A
MES	7.53 (1.01) b,A	10.29 (3.11) a,A	23.3 (1.33) c,A	19 (2.71) c,B	0.99 (0.14) c,d,A	0.76 (0.14) a,b,c,B	24,35 (1.38) b,A	23.56 (1.14) b,A
OMN	18.46 (3.36) c,A	10.87 (2.61) a,B	3.15 (0.57) a,A	4.24 (1.54) a,A	0.55 (0.06) d,e,A	0.66 (0.12) b,A	43.88 (3.96) c,A	38.47 (2.49) c,B
CNA	10.07 (0.47) a, A	7.4 (1.43) b,B	16.73 (0.63) d,A	17.25 (1.37) c,A	0.75 (0.07) a,b,f,A	0.88 (0.09) c,B	24.62 (2.73) b,A	24.81 (1.94) b,A

* The same lower-case letters indicate statistically insignificant differences among materials before or after aging, whereas the same upper-case letters show that the insignificant differences before and after photoaging per material (*p* > 0.05).

**Table 6:** Differences in optical parameters after photoaging (Δ: after–before) *.

Material	ΔL*	Δa*	Δb*	ΔΕ	ΔTP	ΔOP	ΔCR	ΔTT
CHT	−2.56 (−3.67/−1.97) b	0.18 (0.08) b	−0.39 (−0.54/0.03) a	2.8 (0.88) d	−1.65 (−2.31/−0.75) b	−0.02 (0.27) c	0.08 (0.08) a	−4.42 (3.21) a,b,c
ESU	−1.3 (−1.64/−0.48) b	−0.08 (0.19) b	−0.39 (1.49/0.2) a	1.56 (0.3) c,d	0.5 (−1.65/2.71) b	0.71 (0.79) b	−0.01 (0.16) a,b,c,d	−0.62 (1.23) a,b,c
MES	2.58 (−0.46/2.91) a	1.08 (0.96) a	−1.56 (−2.81/0.55) b	3.54 (0.64) b,d	3.32 (0.42/4.82) a	−4.34 (1.99) d	−0.24 (0.15) b	−0.79 (0.54) a,b,c
OMN	−6.16 (−7.57/−5.89) c	−0.03 (0.41) b	−6.59 (−7.79/−3.3) c	8.99 (1.23) a	−7.56 (−8.2/−6.99) d	1.09 (1.41) a	0.11 (0.15) a,c,d	−5.4 (4.34) b
CNA	−2.1 (−3.01/−1.17) b	−1.03 (0.59) c	−3.6 (−4.8/−1.53) d	4.35 (0.81) b	−2.42 (−3.52/−1.95) c	0.52 (1.83) b	0.13 (0.16) a,c,d	−0.19 (2.19) c

* The same lower-case letters indicate mean values and standard deviations (Δa*, ΔE, ΔOP) or median values and interquartile ranges (ΔL*, Δb*, ΔTP, ΔCR, ΔΤΤ) with statistically insignificant differences among materials, whereas the same upper-case letters show the insignificant differences before and after photoaging per material (*p* > 0.05).

**Table 7:** Results of the roughness parameters before (Bf) and after (Af) photoaging at top surfaces *.

Material	Sa Bf (nm)	SaAf (nm)	Sz Bf (μm)	Sz Af (μm)	Sdr Bf (%)	SdrAf (%)	Sds Bf (×10^3^, 1/mm^2^)	Sds Af (×10^3^, 1/mm^2^)	Sc Bf (μm^3^/ mm^2^	Sc Af (μm^3^/ mm^2^)
CHT	70.52 (8.63) a,A	293.98 (28.56) a,B	1.4 (0.59) a,A	4.07 (0.34) a,B	1.23 (1.02/1.62) a,A	40.05 (37.22/44.49) a,B	18.887 (3.687) a,A	37.136 (2.333) a,B	9.2 (1.64) a,A	36.6 (5.27) a,B
ESU	65,13 (8.46) b,A	178.57 (16.58) b,B	1.57 (0.17) a,A	2.31 (0.24) b,B	1.4 (1.01/1.67) a,A	16.12 (13.8/18.02) b,B	16.453 (2.969) a,A	30.235 (1.407) b,e,B	101.4 (22.7) a,A	267.8 (16.96) b,B
MES	116.95 (3.77) b,A	119.44 (17.62) c,A	1.79 (0.33) a,A	2.06 (0.48) b,B	2.71 (2.56/2.88) b,A	3.82 (2.34/4.45) c,A	14.546 (1.484) a,A	18.977 (0.954) c,B	197.6 (10.53) b,A	180.8 (29.5) c,A
OMN	55.77 (3.47) c,A	165.28 (25.03) b,B	0.9 (0.34) b,A	1.91 (10.19) b,B	0.41 (0.35/0.99) c,A	12.3 (10.23/15.57) b,B	24.763 (6.558) b,A	29.635 (1.524) d,e,A	84.6 (7.3) a,A	256.6 (39.9) b,B
CNA	73.82 (16.61) a,A	171.92 (28.59) b,B	1.8 (0.52) a,A	2.28 (0.33) b,B	1.92 (1.21/3.14) a,A	12.54 (11.42/17.55) b,B	15.745 (3.038) a,A	28.099 (2.491) e,B	113.8 (30.28) a,A	253.8 (39.68) b,B

* The same lower-case letters indicate mean values and standard deviations (Sa, Sz, Sds, Sc) or median values and interquartile ranges (Sdr) with statistically insignificant differences among materials, whereas the same upper-case letters show the insignificant differences before and after photoaging per material (*p* > 0.05).

## Data Availability

The original contributions presented in the study are included in the article, further inquiries can be directed to the corresponding author.
